# Assessment of a mass balance equation for estimating community-level prevalence of COVID-19 using wastewater-based epidemiology in a mid-sized city

**DOI:** 10.1038/s41598-022-21354-6

**Published:** 2022-11-09

**Authors:** Andrew L. Rainey, Julia C. Loeb, Sarah E. Robinson, Paul Davis, Song Liang, John A. Lednicky, Eric S. Coker, Tara Sabo-Attwood, Joseph H. Bisesi, Anthony T. Maurelli

**Affiliations:** 1 Department of Environmental and Global Health, College of Public Health and Health Professions, University of Florida, Gainesville, FL 32610 USA; 2 Emerging Pathogens Institute, University of Florida, 2055 Mowry Road, PO Box 100009, Gainesville, FL 32610 USA; 3 Center for Environmental and Human Toxicology, University of Florida, 2187 Mowry Road, PO Box 110885, Gainesville, FL 32611 USA; 4 Gainesville Regional Utilities, Gainesville, FL 32614 USA

**Keywords:** Environmental microbiology, Policy and public health in microbiology

## Abstract

Wastewater-based epidemiology (WBE) has emerged as a valuable epidemiologic tool to detect the presence of pathogens and track disease trends within a community. WBE overcomes some limitations of traditional clinical disease surveillance as it uses pooled samples from the entire community, irrespective of health-seeking behaviors and symptomatic status of infected individuals. WBE has the potential to estimate the number of infections within a community by using a mass balance equation, however, it has yet to be assessed for accuracy. We hypothesized that the mass balance equation-based approach using measured SARS-CoV-2 wastewater concentrations can generate accurate prevalence estimates of COVID-19 within a community. This study encompassed wastewater sampling over a 53-week period during the COVID-19 pandemic in Gainesville, Florida, to assess the ability of the mass balance equation to generate accurate COVID-19 prevalence estimates. The SARS-CoV-2 wastewater concentration showed a significant linear association (Parameter estimate = 39.43, *P* value < 0.0001) with clinically reported COVID-19 cases. Overall, the mass balance equation produced accurate COVID-19 prevalence estimates with a median absolute error of 1.28%, as compared to the clinical reference group. Therefore, the mass balance equation applied to WBE is an effective tool for generating accurate community-level prevalence estimates of COVID-19 to improve community surveillance.

## Introduction

Public health surveillance is used to monitor the health of a community by identifying emerging diseases, estimating the burden of disease, monitoring demographic or spatiotemporal trends, and to evaluate interventions and generate hypotheses for scientific research^1,2^. At the time of writing, severe acute respiratory syndrome coronavirus 2 (SARS-CoV-2), the virus that causes COVID-19, has infected over 30 million people and caused over 800,000 deaths in the United States^3^. The global effort to end the COVID-19 pandemic includes robust community health surveillance to guide implementation of policy and public health control measures to mitigate the spread of SARS-CoV-2^4^. An important principle of COVID-19 surveillance is to estimate disease incidence and prevalence within a community. Such surveillance provides key information that can guide implementation of targeted public health control measures. Individual at-home and clinical testing of blood and nasopharyngeal swabs based on antibody or PCR methods, respectively, severely underestimates the true number of infections within a community due to barriers such as access to testing, health-seeking behavior, and timely reporting of results to health officials^5^. Currently, data from individual-level clinical testing have been used as the primary source for generating community-level estimates^6–9^. This approach is inherently limited in scope and the ability to provide accurate prevalence estimates.

Wastewater-based epidemiology (WBE) is a public health surveillance tool that can aid in assessing the health of a community through analysis of disease-specific biomarkers that are excreted via the urine and feces of infected individuals^10^. Historically, WBE has been successfully utilized to complement clinical surveillance in the global polio eradication efforts by detecting polio virus circulation and outbreaks of polio independent of clinical manifestations of disease in the community^11,12^. While SARS-CoV-2 is a respiratory pathogen, detection of the virus in wastewater is possible because it infects the intestines, and fecal shedding of the virus occurs in both symptomatic and asymptomatic individuals^13–15^. During the COVID-19 pandemic, WBE has been deployed across the globe to track spatiotemporal trends of SARS-CoV-2 infections, identifying potential outbreaks, and monitoring the emergence and circulation of novel viral variants of concern within a community^16–24^. In the United States, WBE of SARS-CoV-2 has been adopted on the national level through the Centers for Disease Control and Prevention (CDC) National Wastewater Surveillance System (NWSS), which was implemented during the COVID-19 pandemic to support community-level pandemic response efforts^25^.

A novel application of WBE is to estimate the prevalence of SARS-CoV-2 infections within a community by using the measured viral concentration in wastewater. A strength of WBE is that it can generate comprehensive population data, as it samples from a pooled community sample with input from symptomatic and asymptomatic individuals. This method overcomes the limitations and biases of traditional clinical surveillance which is dependent on testing individuals and reporting test results. One method that has been proposed for generating a community-level COVID-19 prevalence estimation through wastewater analysis is the mass balance equation^17,18,26,27^. The mass balance equation was originally derived to estimate community-level usage of illicit drugs by measuring drug concentrations in wastewater, individual drug excretion rate, and per capita wastewater flow rate^28,29^. This approach has since been adopted for estimating the community-level prevalence of infectious diseases by measuring pathogen concentration in wastewater, individual-level fecal shedding rate of the pathogen, estimated individual-level daily fecal mass, and wastewater flow rate. Currently, NWSS recognizes that wastewater-derived prevalence estimates could be a “useful application of wastewater surveillance in the future once more is known”^30^. Previous studies have not rigorously assessed this approach with appropriate clinical reference groups or assessed the influence of SARS-CoV-2 fecal shedding on the prevalence outcome produced by the mass balance equation. Thus, there is a need to further assess wastewater-derived prevalence estimates with prevalence estimates derived from clinical surveillance approaches to demonstrate how WBE will advance public health surveillance. If proven to generate accurate prevalence estimates, WBE can improve community level surveillance of diseases that are rare or severely underdiagnosed in the clinical setting.

The objective of this study was to assess the accuracy of the wastewater-derived COVID-19 prevalence estimates generated by the mass balance equation. In order to achieve our objective, we conducted weekly wastewater surveillance of SARS-CoV-2 in Gainesville, FL and utilized the mass balance equation to estimate the weekly community-level prevalence. The city-level prevalence values were compared with the weekly prevalence estimates derived from county-level clinical surveillance data that encompasses our study site. In our study, we found that the mass balance equation produced accurate community-level prevalence estimates compared to clinical surveillance.

## Methods

### Study site and sample collection

**Figure 1:**
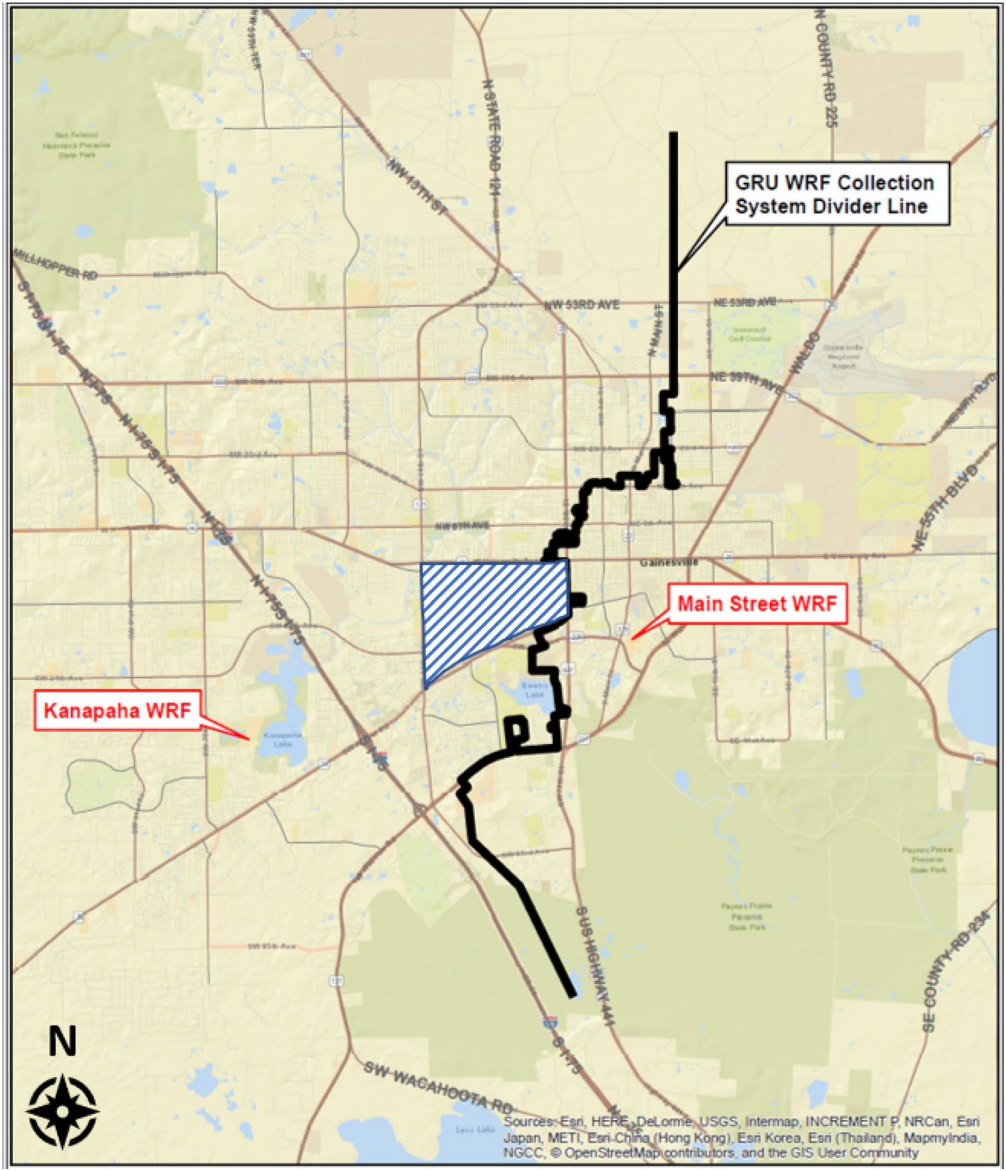
The wastewater service map of Gainesville, FL. The Gainesville Regional Utility (GRU) divider line running through the center of the city separates the service area for the two water reclamation facilities (WRF) that services the east (Main Street WRF) and west (Kanapaha WRF) areas of Gainesville, FL. The shaded blue area in the figure denotes the area of the University of Florida which has an on-campus WRF that independently services the area.

The study was conducted in Gainesville, which is an urban community in North Central Florida (FL) with approximately 133, 997 residents. Gainesville is serviced by a separate (i.e. not mixed with storm water) sanitary sewer system that is connected to two water reclamation facilities (WRF), Kanapaha WRF, which services the west side of the city, and Main Street WRF, which services the east side of the city (Fig. 1).

Fifty-three weekly influent wastewater samples were collected between May 26, 2020 and May 30, 2021 from each WRF for a total of 106 samples. A 24-h composite sample of influent wastewater was collected every Monday at each WRF. The following day, a 1L subsample of each 24-h composite was aliquoted, stored on ice, and transported to our laboratory for analysis. All samples were processed and analyzed within 7 days of collection.

### SARS-CoV-2 concentration and RNA extraction

SARS-CoV-2 RNA was recovered using methods previously described by Rainey et al.^16^. Briefly, we added 500 µL of 2 M MgCl_2_ to a 50 mL sample aliquot and passed the sample through a 0.45 µm pore size mixed cellulose ester membrane filter disc (Millipore Sigma, Burlington, MA, USA). We extracted total nucleic acid from the filter using the Zymo Quick-DNA/RNA Viral MagBead Kit (Zymo Research, Irvine, CA, USA) and further purified the extracted nucleic acid using the Zymo OneStep PCR Inhibitor Removal Kit column (Zymo Research, Irvine, CA, USA). For quality control measures, a negative control filter blank was processed through the concentration and extraction steps and a positive extraction control of RNA from lysed SARS-CoV-2 was used in the extraction step. Final nucleic acid extracts were used for real-time quantitative reverse transcription polymerase chain reaction (rRT-qPCR) analysis.

### SARS-CoV-2 rRT-qPCR

For SARS-CoV-2 detection and quantification, we conducted rRT-qPCR assays of the extracted nucleic acid using the Centers for Disease Control and Prevention (CDC) N2 genetic target^31^. The rRT-qPCR assays were performed using reaction mixtures and cycle conditions previously described^16^. SARS-CoV-2 RNA was quantified using a standard curve ranging from 100,000 to 1.28 (1:5 dilutions) of CDC N2 genomic copies/µL generated by the 2019-nCoV_N_positive control DNA Plasmid from Integrated DNA Technologies (Integrated DNA Technologies, Coralville, IA). The negative filter blank control and positive extraction control were used in each rRT-qPCR assay. The assay limit of detection was set at a cycle threshold (Cq) cutoff value ≤ 40 cycles to determine a positive sample. The assay limit of quantification of 32 genomic copies/5 µL was the lowest detectable dilution concentration from the standard curve. Assays were included in data analysis if the standard curve amplification efficiency was within the accepted range of 90–110%, and if the assay passed all quality control measures. Quantifiable samples were back calculated to Log_10_ genomic copies/L (GC/L). The quantified value for both WRFs were summed to represent the overall city Log_10_ GC/L. Positive and quantifiable wastewater sample results were reported as a concentration representing an entire week (Monday–Sunday) for analysis with weekly COVID-19 clinical incidence and prevalence (see below).

### COVID-19 clinical incidence and prevalence

COVID-19 case data were obtained from the Florida Department of Health (FL DOH) COVID-19 Daily Case and Monitoring State Reports^32^. The COVID-19 city case data for Gainesville represents clinically confirmed COVID-19 cases of FL residents based on the patient's zone improvement plan (ZIP) Code. Positive cases were identified from approved PCR, antigen, or antibody diagnostic test results reported to the FL DOH from the state public health laboratories and commercial and hospital laboratories. Daily reported COVID-19 cases were summed (Monday–Sunday) to generate the weekly COVID-19 incidence in Gainesville, FL. Our study period ended May 30, 2021 due to a switch of COVID-19 data reporting techniques by the FL DOH^33^. On June 3, 2021, the FL DOH began to only report weekly case incidence on the county- and state-level, but not on the city-level across the entire state. Due to this significant change and lack of consistency in data reporting, we were no longer able to reliably track the COVID-19 case incidence in Gainesville, FL.

The test positivity rate was used as a reference indicator of COVID-19 clinical prevalence in our study population, as this measurement has been previously used for generating generalizable community-level COVID-19 prevalence estimates^24,34–38^. The current "gold standard” approach for obtaining a community-level prevalence with the test positivity rate is through individual testing with randomized community surveys^35^. Logistically, this was not possible for our study, so we used the community-level test positivity rate obtained through clinical testing that was reported through the FL DOH. The test positivity rate data were obtained from the FL DOH COVID-19 Daily Case and Monitoring County Reports^32^. We used the county-level (Alachua County, FL) data for the test positivity rate because FL DOH did not report these data on the city level. The daily test positivity rate for Alachua County represents the number of people that test positive divided by all the people tested that day. This number excludes people who have previously tested positive but does not exclude people who have previously tested negative. The daily reported test positivity rate (Monday–Sunday) was converted to the average weekly COVID-19 clinical prevalence in Alachua County.

To support the use of the county-level test positive rate for a city-level study, we compared the COVID-19 case incidence from the city and the county to determine if the county-level data were largely representative and generalizable to the city. We found that the weekly COVID-19 case incidence in Gainesville, FL accounted for an average of 77% of the weekly Alachua County COVID-19 case incidence, and that the city-level cases displayed an extremely strong correlation with the county-level cases (R = 0.99) (Supplemental Fig. 1). The results support the representativeness and generalizability of the county-level data for use of our study in Gainesville, FL. We assessed the linear association of the quantified SARS-CoV-2 wastewater concentrations with the weekly COVID-19 incidence and weekly COVID-19 clinical prevalence. In addition to linear regression, we further assessed the linearity of these associations by converting the SARS-CoV-2 wastewater concentrations into tertiles and performing Poisson regression. All regression analyses employed a *P* value significance level of α = 0.05.

### Mass balance equation

The wastewater-derived prevalence of COVID-19 within a community was estimated using the mass balance equation expressed in Eq. (1). *W* is the measured concentration of viral RNA genome copies (GC) per liter wastewater from the qRT-PCR analysis for each wastewater sample (Log_10_ SARS-CoV-2 GC/L); *F* is the average flow rate of the influent wastewater measured at the WRF for each day when the 24-h composite wastewater sample was collected (L/day); *G* is a constant value of the average mass of feces a person produces each day (128 g of feces per person/day), as determined from Rose et al.^39^; Parameter *I* is the SARS-CoV-2 fecal shedding rate (Log_10_ SARS-CoV-2 GC/gram of feces); and *N* is the total number of SARS-CoV-2 infected individuals contributing to the wastewater sample. The SARS-CoV-2 fecal shedding rate (*I*) is a parameter of high variability as previous studies that have measured the viral load of SARS-CoV-2 in the feces of infected individuals show a broad range in the average fecal shedding concentration^13,40,41^. To calculate prevalence, we divide the total number of SARS-CoV-2 infected individuals (*N*) over the 2019 United States census population of Gainesville, FL (133, 997).

1 $$\frac{W \times F }{G \times I }=N$$

### Statistical analysis

We analyzed the association of the weekly COVID-19 clinical incidence with the weekly COVID-19 clinical prevalence using a univariate linear regression model. We then used a univariate linear regression model to analyze the city SARS-CoV-2 RNA wastewater concentrations with the weekly COVID-19 clinical incidence and prevalence. Univariate linear regression model *P* values were calculated for a 2-tailed test, and a confidence level of alpha = 0.05 was used to reject the null hypothesis.

Due to the high level of variability in the reported SARS-CoV-2 fecal shedding rate (*I*), we conducted a sensitivity analysis to examine the influence of this variability on the COVID-19 prevalence estimates generated from the mass balance equation. We are specifically investigating the influence of a point estimate of the average reported fecal shedding rate, and not the impact of variable shedding rates from an individual over the course of infection. We generated the weekly wastewater-derived prevalence estimates for three models that use a representative range of SARS-CoV-2 fecal shedding rates reported from Gerrity et al. (8.9 Log_10_ GC/g feces) (Model A)^17^, Lescure et al. (6.8 Log_10_ GC/g feces) (Model B)^40^, and Miura et al. (3.4 Log_10_ GC/g feces) (Model C)^41^. We compared the difference of the prevalence estimates generated from each model to assess the influence of the SARS-CoV-2 fecal shedding rate on the mass balance equation. We first performed a nonparametric Kruskal–Wallis test among all of the models, and then conducted a Dwass, Steel, Critchlow–Fligner test for pairwise comparisons between each of the models with a *P* value significance level of α = 0.05.

To assess the accuracy of the wastewater-derived prevalence estimates, we calculated the median absolute error (MAE) and median absolute percentage error (MAPE), which is a test that has been previously conducted in similar WBE studies^36,42^. The MAE is the absolute difference in the prevalence estimates from each wastewater model, as compared to our clinical reference group. Since the MAE is already reported as a percent difference, it is important to note that the MAPE represents that absolute error percent difference as a proportion of the clinical reference group. The distribution of the absolute differences was assessed with the Shapiro–Wilk test and the data was determined to have a non-normal distribution (*P* value < 0.05), so the median statistic was reported in the analysis because it can be less prone to influence from outliers. All statistical analyses were performed in SAS version 9.4 software (SAS Institute, Inc, Cary, North Carolina). All figures were generated using JMP Pro (SAS Institute, Inc, Cary, North Carolina).

## Results

### COVID-19 clinical incidence and prevalence

**Figure 2:**
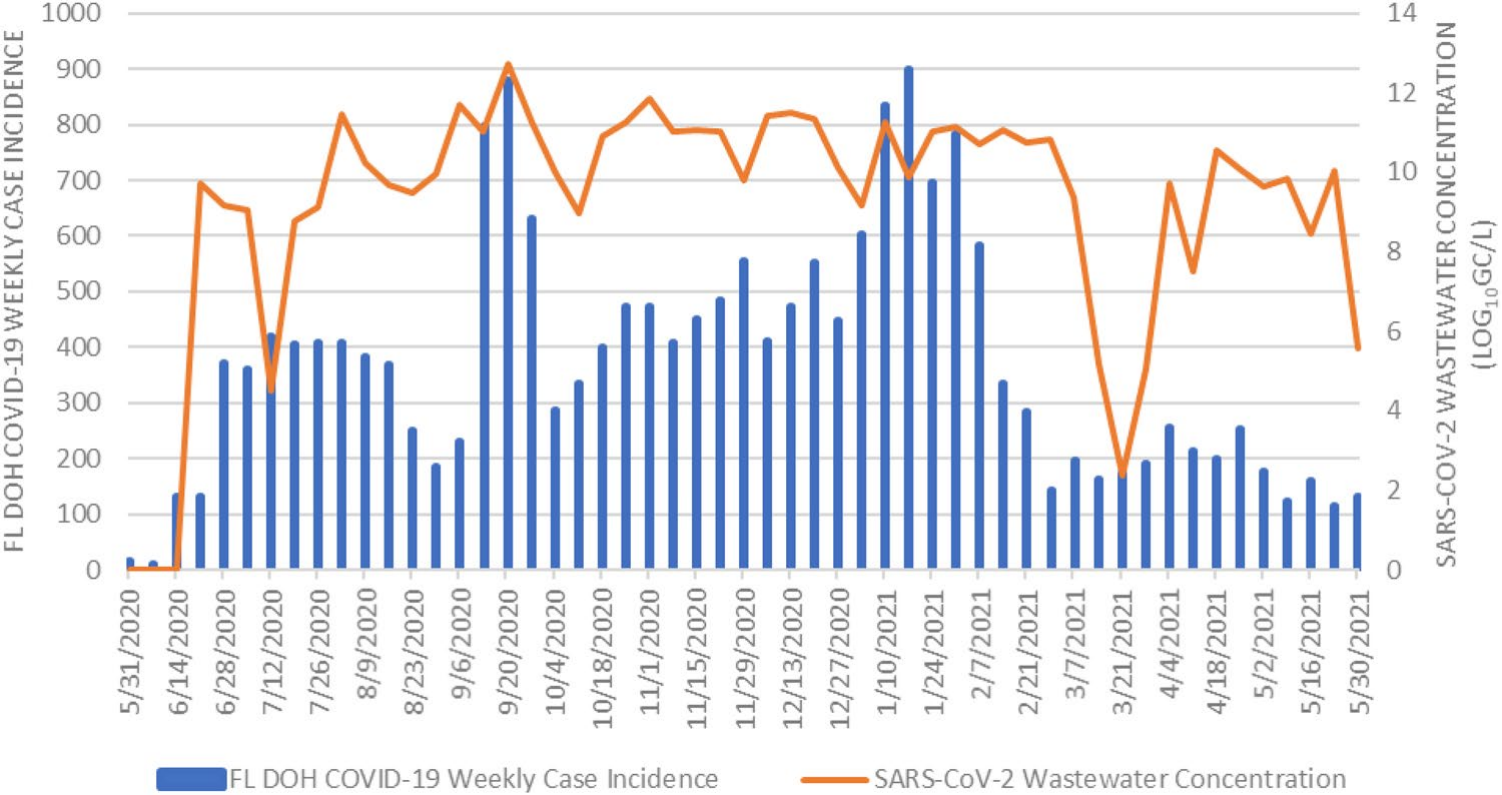
Weekly COVID-19 clinical case incidence and overall SARS-CoV-2 wastewater concentration (Log_10_ GC/L) in Gainesville, FL between May 26, 2020 and May 30, 2021.

The weekly counts of the COVID-19 clinical incidence are featured in Fig. 2. Over the 53-week study period there was a total of 19,631 COVID-19 cases reported in the city of Gainesville with a mean of 370 COVID-19 cases reported each week (range 9–898 cases). The two largest peaks of COVID-19 incidence occurred during the weeks of September 20, 2020 (880 cases) and January 17, 2021 (898 cases). The mean weekly COVID-19 clinical prevalence during the study period was 5.13% (range 0.26–14.67%).

### Measurement of SARS-CoV-2 RNA in wastewater by rRT-qPCR

All rRT-qPCR assays passed quality control measures and were accepted for data analysis. The SARS-CoV-2 wastewater concentrations in the city over our study period are shown in Fig. 2. The mean SARS-CoV-2 wastewater concentration in Gainesville, FL over the study period was 9.19 Log_10_ GC/L (range 0–12.73 Log_10_ GC/L). Among the 53 total weeks that were sampled in this study, 50 weeks yielded a positive and quantifiable result for the presence of SARS-CoV-2 RNA. The first week to yield a positive wastewater sample was June 16, 2020, the fourth week of sampling in this study. The final week sampled in our study, May 25, 2021, also tested positive for SARS-CoV-2.

### Linear association of the SARS-CoV-2 wastewater concentrations and COVID-19 clinical outcomes

**Figure 3:**
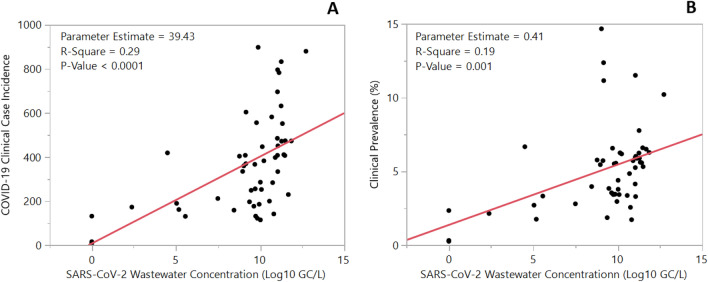
Linear association of the SARS-CoV-2 wastewater concentration (Log_10_ GC/L) and weekly COVID-19 clinical incidence (**A**) and weekly COVID-19 clinical prevalence (**B**) between May 26, 2020 and May 30, 2021 in Gainesville, FL.

Linear regression analysis showed a Log_10_ GC/L unit increase of the weekly wastewater SARS-CoV-2 concentration was positively associated with an increase in the weekly COVID-19 incidence by 39.43 cases (*P* < 0.0001; R-Square, 0.29) (Fig. 3). The Poisson regression test for linearity between SARS-CoV-2 wastewater concentrations and weekly COVID-19 incidence showed a significant increase among wastewater concentration tertiles (*P* < 0.0001) (Table 1). Linear regression analysis showed that as the weekly COVID-19 incidence increased by 100 cases, the weekly COVID-19 clinical prevalence increased by 0.82% (*P* < 0.001; R-Square, 0.402). Linear regression analysis among the clinical incidence and clinical prevalence showed that a Log_10_ GC/L unit increase of the weekly SARS-CoV-2 wastewater concentration was positively associated with a 0.41% increase of the COVID-19 clinical prevalence (*P* = 0.0012; R-Square, 0.19) (Fig. 3). However, the Poisson regression test for linearity between SARS-CoV-2 wastewater concentrations and weekly COVID-19 clinical prevalence showed no significant association among wastewater concentration tertiles (*P* > 0.05) (Table 1).

**Table 1:** Test of linearity among SARS-CoV-2 wastewater concentrations and COVID-19 clinical outcomes.

Wastewater concentration group	Clinical incidence parameter estimate (standard error)	Clinical incidence *P* value	Clinical prevalence parameter estimate (standard error)	Clinical prevalence *P* value
0 (Reference) (range = 0–9.37 Log_10_GC/L)	–		–	
1 (range = 9.47–10.81 Log_10_GC/L)	0.22 (0.02)	< 0.0001	− 0.16 (0.16)	0.30
2 (range = 10.90–12.73 Log_10_GC/L)	0.76 (0.02)	< 0.0001	0.26 (0.14)	0.08

Poisson regression analysis of SARS-CoV-2 wastewater concentration tertiles and weekly COVID-19 clinical incidence and clinical prevalence.

### Mass balance equation COVID-19 prevalence estimates

The median weekly wastewater-derived COVID-19 prevalence as calculated by the mass balance equation was 4.51% (range 0–7.07%) (Model A), 5.90% (range 0–9.26%) (Model B), and 11.81% (range 0–18.52%) (Model C) (Supplemental Fig. 2). There was a significant difference among all models of the wastewater-derived COVID-19 prevalence estimates (Chi-Square, 75.03; *P* value < 0.0001). The Dwass, Steel, Critchlow–Fligner test showed a significant difference in the pairwise model comparison of the wastewater-derived COVID-19 estimates (*P* value < 0.0001) (Supplemental Table 1). The sensitivity analysis showed that parameter *I* (the SARS-CoV-2 fecal shedding rate) has a significant influence over the outcome produced by the mass balance equation. This result provided a justification to assess all three wastewater models in the final analysis to compare the wastewater prevalence estimates with the clinical prevalence reference group.

**Figure 4:**
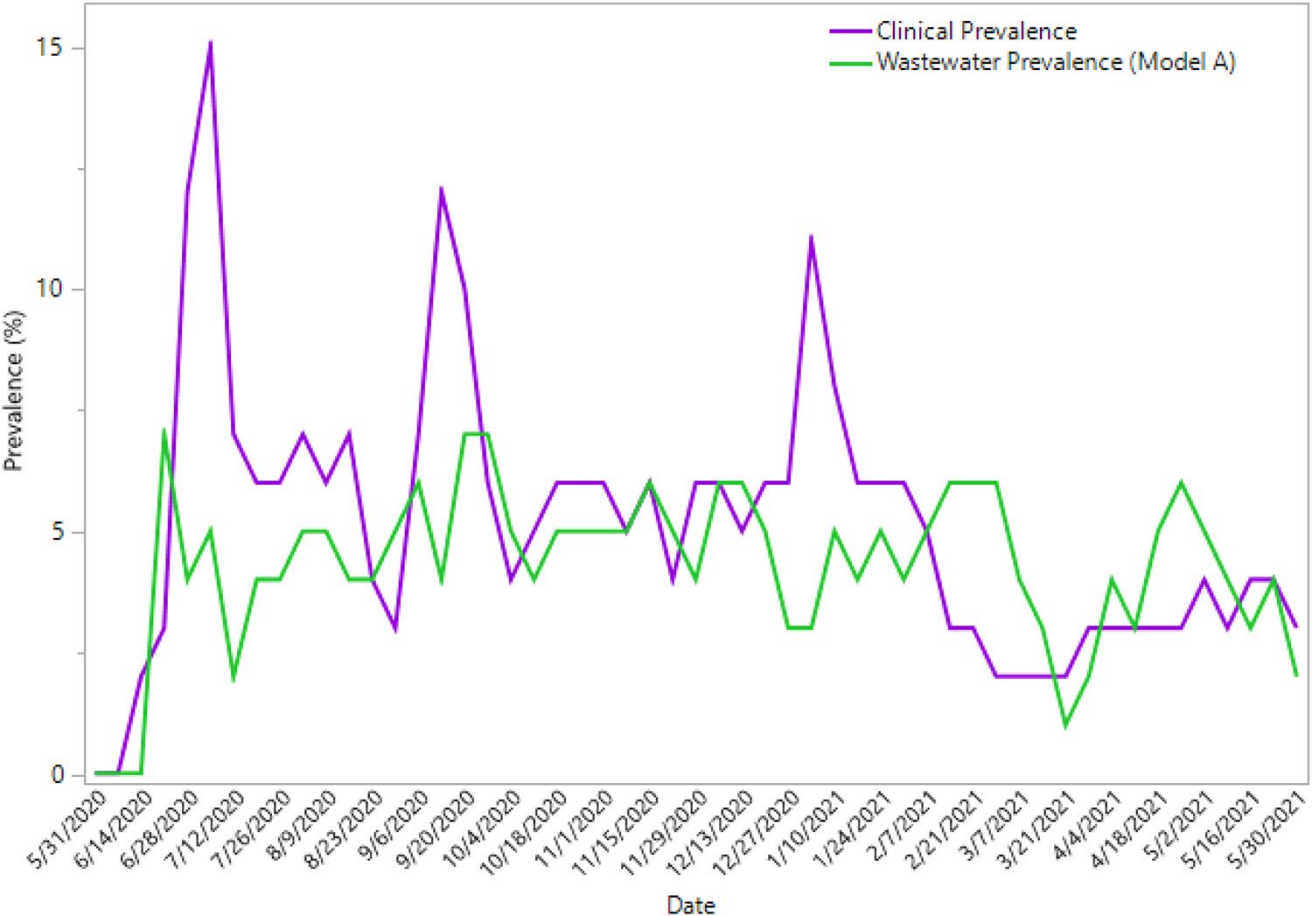
Estimated COVID-19 prevalence estimates between May 26, 2020 and May 30, 2021 in Gainesville, FL. The COVID-19 clinical prevalence is derived from the FL DOH weekly test positivity rate and the Model A (*I* = 8.9 Log_10_ GC/g feces) wastewater prevalence was calculated by using the mass balance equation.

Weekly wastewater-derived COVID-19 prevalence estimate models over our study period produced estimates that were both slightly higher and lower than the COVID-19 clinical prevalence estimates (Table 2). The MAE and MAPE for each wastewater model can also be found in Table 2. The MAE and MAPE of Model A were the best of all three wastewater models at 1.28 (MAE) and 28.77 (MAPE). The MAE and MAPE of Model B were slightly higher than Model A, at 1.54 (MAE) and 40.54 (MAPE). Model C had the highest MAE and MAPE of 6.24 and 113.80, respectively. The city-level COVID-19 prevalence estimates generated from Model A were not significantly different from our reference group. The MAE and MAPE of Model A were also the lowest, indicating that Model A was the best performing model in our study by generating accurate prevalence estimates. The weekly COVID-19 clinical prevalence and Model A wastewater prevalence trends can be found in Fig. 4.

**Table 2:** Difference between wastewater-derived COVID-19 prevalence and clinical prevalence between May 26, 2020 and May 30, 2021 in Gainesville, FL.

Prevalence source	Median absolute error (MAE)	Median absolute percentage error (MAPE)
Clinical (Reference)	–	–
Wastewater (Model A)	1.28	28.77
Wastewater (Model B)	1.54	40.54
Wastewater (Model C)	6.24	113.80

Median Absolute Error (MAE) and median absolute percentage error (MAPE) of the difference between the wastewater-derived COVID-19 prevalence estimate and the clinical prevalence (reference group).

## Discussion

The objective of our study was to apply the mass balance equation to weekly wastewater surveillance (May 2020–May 2021) of SARS-CoV-2 to estimate the weekly community-level prevalence of COVID-19, and to assess the equation by comparing the wastewater-derived COVID-19 prevalence estimates with the weekly COVID-19 prevalence estimate derived from clinical surveillance data that encompassed the same community. We were also able to measure the influence of the SARS-CoV-2 fecal shedding rate parameter on the COVID-19 prevalence produced from the mass balance equation. From our assessment, the mass balance equation produced COVID-19 prevalence estimates that were less than 1% different than our clinical reference group; demonstrating that the mass balance equation is an effective approach for estimating the prevalence of COVID-19 within a community.

Wastewater surveillance is a useful tool that can generate data for researchers and public health officials to help them better understand the ever-changing dynamics of COVID-19 within a community. Our weekly wastewater surveillance measurements of SARS-CoV-2 were able to help identify community dynamics of COVID-19 over a 53-week period in Gainesville, FL. In our study, the highest peak in the SARS-CoV-2 wastewater concentration occurred in September 2020 (12.73 Log_10_ GC/L) (Fig. 2). This peak coincides with the reintroduction of a large population of university students to the University of Florida for the start of the Fall academic semester. The university is host to more than 50,000 students, adding a large number of potentially susceptible individuals to the community that may be the source of an increase in SARS-CoV-2 infections, as detected by our wastewater surveillance. Two weeks with the lowest reported case incidences occurred during the breaks in between the Spring and Summer academic semesters at the university where there were no classes in session. Both of these results suggest a major influence of the university population on the burden of COVID-19 in the city. There was also a sharp peak in the COVID-19 clinical prevalence during the week of January 3, 2021 while there was a decrease in the wastewater-derived COVID-19 prevalence (Fig. 4). This sharp increase in the clinical prevalence was identified as an outlier by the FL DOH. They noted that testing was limited and often not available due to holidays which “resulted in less people tested and delays in result processing and reporting which have impacted Florida’s daily testing number by nearly half”^43^. There was a decrease in the SARS-CoV-2 wastewater concentrations in March 2021 that resulted in the lowest quantifiable wastewater concentration in our study period (2.38 Log_10_ GC/L) (Fig. 2). This sharp decrease coincided with a low number in the reported weekly COVID-19 case incidence. During this time, COVID-19 vaccines were becoming more available to the general public and more than 60,000 individuals had been vaccinated in Alachua County where Gainesville, FL is located. In addition to our study, other reports have shown that the detection of SARS-CoV-2 in wastewater decreases as community vaccinations increase, showing how wastewater surveillance is also an effective approach for monitoring public health interventions, such as COVID-19 vaccinations^25,44^. WBE can continue to provide real-time community-level disease trends, regardless of individual test-seeking behaviors, that can be used to better understand community-level disease dynamics that can be leveraged in policy decision making by local public health officials.

Numerous studies across the globe have utilized WBE in various community settings throughout the COVID-19 pandemic^17–24,38,45–48^. Many have shown that trends in SARS-CoV-2 wastewater concentrations reflect the clinical case incidence of COVID-19, however, more details are needed to better understand these trends^38,45–49^. Firstly, previous studies have recommended a sampling frequency of at least two times per week to obtain significant real-time relationships, with a stronger association (R-Square value) between the SARS-CoV-2 wastewater concentration and COVID-19 cases^46,50^. In our study, we sampled once per week and were still able to obtain a significant linear association (R-Square = 0.29) with the wastewater concentrations and weekly COVID-19 case incidence (Fig. 3A). Furthermore, there was a significant linear association between the SARS-CoV-2 wastewater concentration with the COVID-19 clinical prevalence (Fig. 3B); however, an additional test of linearity showed there was not a significant linear association (Table 1). Stronger correlations with the COVID-19 case incidence have been reported from multiple other studies with correlations above 0.90^38,46,51,52^. A previous study from Larsen et al. showed a significant association (R-Square = 0.55) with the test positivity rate with a sampling frequency of at least twice per week^53^. We believe that the strength of the linear associations in our study would have been higher with an increase in our sampling frequency.

Currently, there is no consensus of the SARS-CoV-2 *I* parameter (SARS-CoV-2 fecal shedding rate) estimate. The current published literature provides a broad range of reported values which can have a significant influence on the outcomes produced by the mass balance equation^18,41,54^. Thus, we believed it was important to investigate the influence of different parameter estimates on the COVID-19 prevalence produced from the mass balance equation. Our analysis, which used values that were representative of the broad range of reported *I* values from published literature, resulted in significant different COVID-19 prevalence estimates with the mass balance equation (Fig. 4). We determined that the *I* parameter estimate used in Model A (*I* = 8.9 Log_10_ GC/g feces) produced the most accurate COVID-19 prevalence estimates (Table 2). McMahan et al. obtained similar results using the mass balance equation with a fixed parameter estimate for *I* of 7.7 Log_10_ GC/g feces to estimate the prevalence of COVID-19 within their study community^26^. In a previous study, Li et al. suggested that the uncertainties from the *I* parameter in the mass balance equation could generate inaccurate prevalence estimates^51^. However, our study and the findings from McMahan et al.^26^, support the use of the mass balance equation as it can produce accurate COVID-19 prevalence estimates.

It is important to note that the fecal shedding rates used in our study did not consider changes of the rate due to the introduction of new viral variants into the community. Some evidence suggests a difference in the individual fecal shedding rate is associated with newly introduced viral variants to a community^55–58^. In addition to differences in the fecal shedding rate due to a new viral variant, Prasek et al. also reported differences in the fecal shedding rate due to population demographics^58^. While the shedding rate may change when a new viral variant is introduced into a community, additional research and analysis is needed to determine if those changes significantly impact COVID-19 infection estimates from wastewater surveillance data.

While the mass balance equation has been used previously to estimate the number of SARS-CoV-2 infected individuals within a community, we aimed to conduct a thorough assessment to determine if it could produce accurate COVID-19 prevalence estimates within a community. From our assessment, we demonstrated that the mass balance equation can produce weekly COVID-19 prevalence estimates with a weekly difference of less than 1% (using Model A) with a MAPE of 28.77 from our real-time COVID-19 clinical prevalence reference group (Table 2). Our results further support conclusions from previous research which used the mass balance equation to provide accurate estimates of the number of infections in a community^17,18,26,27^. While our study has shown that this simplified equation is accurate, numerous studies have utilized more complex, non-linear models for estimating the number of infected individuals in a community using wastewater surveillance data^36,42,51,59,60^. These studies also demonstrated the accuracy of their models when compared to clinical COVID-19 case estimates. One such study from Cao and Francis that utilized a Vector Autoregression (VAR) model across three communities, reported MAPE values ranging from 11 to 58%^42^. While a complex model such as VAR can perform well, the MAPE obtained from Model A in our study (28.77) shows that the simpler mass balance equation can be just as effective at generating COVID-19 prevalence estimates. Importantly, one study by Vallejo et al., compared a simple linear model to a more complex and non-linear model and reported that the non-linear model performed the best. However, they noted that the linear model produced similar results and suggested that the benefit of simplicity provides an advantage that should not be overlooked when deciding on which approach to use within a community^59^. While more complex models may seem to provide more precise estimates, a much simpler model such as the mass balance equation can still provide accurate prevalence estimates and can be used by communities, especially those in low resource settings that may not have access to, or the capabilities to perform, such computing techniques.

In order to establish the real-world application of the mass balance equation to assess the COVID-19 prevalence, it was important to select an accurate reference group for comparison. A key limitation in any approach is that there will never be a perfect comparison group, as the true prevalence of disease in a community cannot be practically obtained. The underestimation of COVID-19 cases from traditional clinical surveillance can make it difficult to identify an appropriate reference group. This can be observed in our study, where a limitation in our clinical data was that the FL DOH excluded case counts for individuals who previously tested positive, meaning that individuals who had a prolonged infection (and still shedding SARS-CoV-2 in wastewater) were not accounted for across multiple weeks. This is a limitation that contributes to further underestimations of clinical COVID-19 cases in our study. A previous study by McMahon et al. that assessed the mass balance equation compared the wastewater-derived COVID-19 estimates in their community to a fixed multiplier of 15 for their clinical prevalence reference group, i.e. the ratio of “true” COVID-19 cases for every one reported COVID-19 clinical cases in their study community^26^. The correction factor is a potential limitation in their study because COVID-19 community testing is dynamic, and any fluctuations in testing frequency during the pandemic would lead to the need for different clinical case multipliers. Numerous studies have used an estimated prevalence from the test positive rate, either from small community-level random sampling or from broader community testing efforts^34–37,51,53,60^. Specifically, Layton et al. were able to conduct small-scale community seroprevalence testing for comparison to their wastewater-derived prevalence estimates^36^. While this is an optimal technique, logistical and economic constraints associated with this approach have been previously noted^61^. Even with the added benefit of small-scale community testing for estimating the COVID-19 prevalence, it still has its own limitations with reporting bias and false negatives reported with individual clinical tests that were used (PCR and antigen testing)^37^. These inherent limitations in community surveillance using clinical testing approaches show the importance of finding additional complementary community surveillance approaches, such as WBE.

In our study, we used the county-level test positivity rate as our real-time (i.e., weekly) COVID-19 clinical prevalence values for our comparison against our wastewater-derived COVID-19 prevalence estimates. The use of county COVID-19 prevalence estimate data for city-level wastewater surveillance data is an important limitation of our study. We attempted to overcome this limitation by showing that the COVID-19 case incidence in Gainesville represented 77% of the total number of cases in the county case incidence, and that the weekly case incidence in Gainesville and Alachua County displayed an extremely high correlation (R = 0.99) with each other (Supplemental Fig. 2). Our study period occurred several months into the COVID-19 pandemic, where community level testing and access to testing sites were operating at its highest capacity. We believe the use of the test positive rate as a real-time clinical reference was appropriate for our study and the test positive rate may be useful in future WBE studies that aim to assess models that estimate the COVID-19 incidence or prevalence within a community. However, another potential limitation to consider is that as both COVID-19 testing capacity and access throughout a community decrease, the test positivity rate may lose its representativeness and generalizability to the broader community. Our study has provided evidence that application of the mass balance equation can improve WBE and community surveillance of COVID-19.

## Conclusions

The COVID-19 pandemic revealed the need for improved community surveillance efforts to better understand disease dynamics to guide public health policy. WBE was adopted early in the pandemic across the globe to fill this need by monitoring viral trends, predicting spikes in hospitalizations, and tracking viral variants circulating within a community^21,38,42,46,47,49,62–65^. In this study we assessed the mass balance equation using WBE and determined that it is a useful and effective tool for generating accurate COVID-19 prevalence estimates within a community. In addition to COVID-19, WBE can be applied to other disease-specific biomarkers that are excreted via urine and feces to monitor other diseases such as Norovirus or Salmonellosis. Use of the mass balance equation and expansion of WBE to other infectious diseases can equip public health and local government officials to better track infection trends and identify potential outbreaks for timely messaging and interventions throughout a community. Overall, our study has generated evidence in support of the CDC National Wastewater Surveillance System effort to provide robust national wastewater surveillance to improve community health.

## Supplementary Information

Supplementary Information.

## Data Availability

Data supporting the results reported in the article can be obtained by contacting the corresponding author A.T.M.
