# Supplementary material for: Assessment of a mass balance equation for estimating community-level prevalence of COVID-19 using wastewater-based epidemiology in a mid-sized city
Source: Sci Rep. 2022 Nov 9;12:19085. doi: 10.1038/s41598-022-21354-6 (PMC9645338; doi:10.1038/s41598-022-21354-6)
Supplement: Supplementary file 1 — Supplementary Information. [file 41598_2022_21354_MOESM1_ESM.docx]

***Supplemental Tables and Figures***

*Supplemental Table 1****:*** Dwass, Steel, Critchlow-Fligner (DSCF) Test of wastewater-derived weekly COVID-19 prevalence estimates. A pairwise analysis, including the difference in the mean of the wastewater-derived prevalence generated by each model of the mass balance equation.

| **Wastewater Model** | **Difference** | **DSCF Value** | **P-Value** |
| --- | --- | --- | --- |
| A - B | -1.32 | 6.26 | <0.0001 |
| A - C | -6.92 | 10.29 | <0.0001 |
| B - C | -5.60 | 9.45 | <0.0001 |

*Supplemental Figure 1:* Pearson’s correlation test of the weekly city-level COVID-19 clinical case incidence in Gainesville, FL with the weekly county-level COVID-19 clinical case incidence in Alachua County, FL.

***
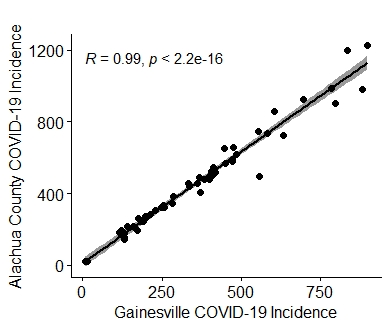
***

*Supplemental Figure 2:* Wastewater-derived COVID-19 prevalence (%) estimates between May 26, 2020 and May 30, 2021 in Gainesville, FL. Box plots of the distribution of the prevalence for the COVID-19 prevalence generated from Models A-C of the mass balance equation. ANOVA analysis of the models with the reported Kruskal Wallis Chi-Square statistic and p-value.
